# An Uncommon Cause of Respiratory Distress: Ruptured Tension Gastrothorax

**DOI:** 10.7759/cureus.87000

**Published:** 2025-06-29

**Authors:** Kourtney Monk, Maria Enders, Lindsay Maguire

**Affiliations:** 1 Emergency Medicine, University of Kansas School of Medicine, Wichita, USA; 2 Emergency Medicine, University of Kansas Medical Center, Kansas City, USA

**Keywords:** bilothorax, cardiothoracic imaging, cardiothoracic surgeries, emergency medicine, gastrothorax, respiratory distress

## Abstract

A gastrothorax occurs when the stomach becomes displaced into the thoracic cavity. Rarely, this gastrothorax can rupture, which can lead to respiratory distress, hemodynamic compromise, and cardiac arrest. Here, we discuss the case of a 69-year-old male who presented to the emergency department (ED) for evaluation of generalized weakness and shortness of breath without associated upper respiratory symptoms. On physical exam, the patient was hemodynamically unstable with absent lung sounds on the left. A portable chest X-ray (CXR) was obtained, which initially revealed concern for tension hydropneumothorax. A chest tube was placed, which drained a large amount of bilious fluid and stabilized the patient. CT imaging revealed a large diaphragmatic hernia with herniation of the stomach into the left hemithorax and a chest tube coursing lateral to and above the stomach. The patient was taken emergently to the operating room, where a large area of necrosis was noted about the greater curvature of the stomach with ischemic perforation. The patient underwent partial gastrectomy and primary diaphragmatic hernia repair and recovered despite a complicated hospital course. Ruptured gastrothorax is rare and presents similarly to other causes of tension physiology. Clinicians should consider CT imaging in stable patients when diaphragmatic hernia or ruptured gastrothorax is possible, and should consider placing a chest tube above the level of the stomach if they suspect this diagnosis by history and CXR in an unstable patient.

## Introduction

A diaphragmatic hernia is a defect in the diaphragm through which abdominal contents can pass into the thorax. These defects are most commonly congenital and diagnosed via antenatal scans, but can be acquired [1]. During fetal development, large defects cause pulmonary hypoplasia, respiratory distress, and potential newborn demise [2]. If the defect is minimal, patients may remain asymptomatic. It is estimated that up to 6% of the adult population has a congenital diaphragmatic hernia (CDH), but the exact prevalence is unknown [3]. Left-sided defects are more common due to later fusion of the diaphragmatic clefts during development [4]. Omentum (92%) and colon (58%) are the most likely structures to herniate, but the stomach can as well (25%), which leads to a gastrothorax. In adults, these herniations occur as a result of increased intra-abdominal pressure, such as from obesity, pregnancy, or trauma [5]. Many cases remain benign; however, some herniations can cause tissue strangulation, necrosis, or even organ rupture [6], leading to accumulation of intraluminal contents and increased intrathoracic pressure, which can cause respiratory distress and hemodynamic compromise [5]. Traumatic diaphragmatic hernias are less common than CHDs, and incidence is estimated to be 0.8-5% in patients with multiple traumatic injuries [7].

Despite the relative commonness of diaphragmatic hernias, intrathoracic gastric perforation is rare, with few cases reported in the literature [8-10]. Mortality after intrathoracic gastric perforation has been suggested to approach 50% [8].

In this article, we discuss the case of a patient with a previously asymptomatic fat-containing diaphragmatic hernia who presented to the emergency department (ED) with respiratory distress and hemodynamic instability resulting from a gastrothorax that had ruptured secondary to tissue ischemia, requiring emergency intervention. This case was previously published as a brief abstract in the Kansas Journal of Medicine. 

## Case presentation

A 69-year-old male with a medical history including paroxysmal atrial fibrillation, hypertension, and diffuse idiopathic skeletal hyperostosis presented for evaluation of generalized weakness for two days and respiratory distress. He noted that his respiratory difficulty began immediately after sneezing. He denied cough. The patient noted a single episode of emesis but denied any ongoing nausea, abdominal pain, chest pain, or fevers. He denied any recent trauma. He was evaluated in the ED the day prior for opioid withdrawal due to abrupt discontinuation of his fentanyl patches, but he was otherwise stable at that time. 

On exam, the vital signs revealed a temperature of 100.1°F, hypotension (90/69), tachycardia (165 bpm), tachypnea (50), and hypoxia (88% on 15 L non-rebreather). The patient was alert but toxic-appearing with dry mucous membranes. Cardiac auscultation revealed irregular tachycardia. He was in respiratory distress: tachypneic with supraclavicular retractions and absent breath sounds throughout the left lung field. His abdomen was soft but mildly distended and tender to palpation in the left upper quadrant. 

Relevant laboratory data are listed in Table 1. The remainder of the patient's laboratory studies, including electrolytes, liver function, and thyroid panel, were unremarkable. Lactic acid improved to 4.6 mM after 30 cc/kg fluid resuscitation. High-sensitivity troponin trended up to 529 ng/L after two hours. Blood cultures were drawn, and no growth was noted.

**Table 1:** Emergency department laboratory values RI: reference interval

Test Name	Patient Result	Reference Range	Unit
White Blood Cells (WBC)	33.3	4.5-11.0	K/uL
Hemoglobin	13.2	13.5-16.5	g/dL
Platelet Count	226	150-400	K/uL
Neutrophils	82	41-77	%
Monocyte Distribution Width (MDW)	26	<20.7	RI
Carbon Dioxide (CO_2_)	15	21-30	mmol/L
Anion Gap	24	3-12	RI
Lactate	11.3	0.5-2.0	mmol/L
Creatinine	1.86	0.4-1.24	mg/dL
Glucose	201	70-100	mg/dL
High Sensitivity Troponin	338	<20	ng/dL
N-Terminal-Pro-B Natriuretic Peptide (BNP)	6260	<125	pg/mL

**Figure 1:**
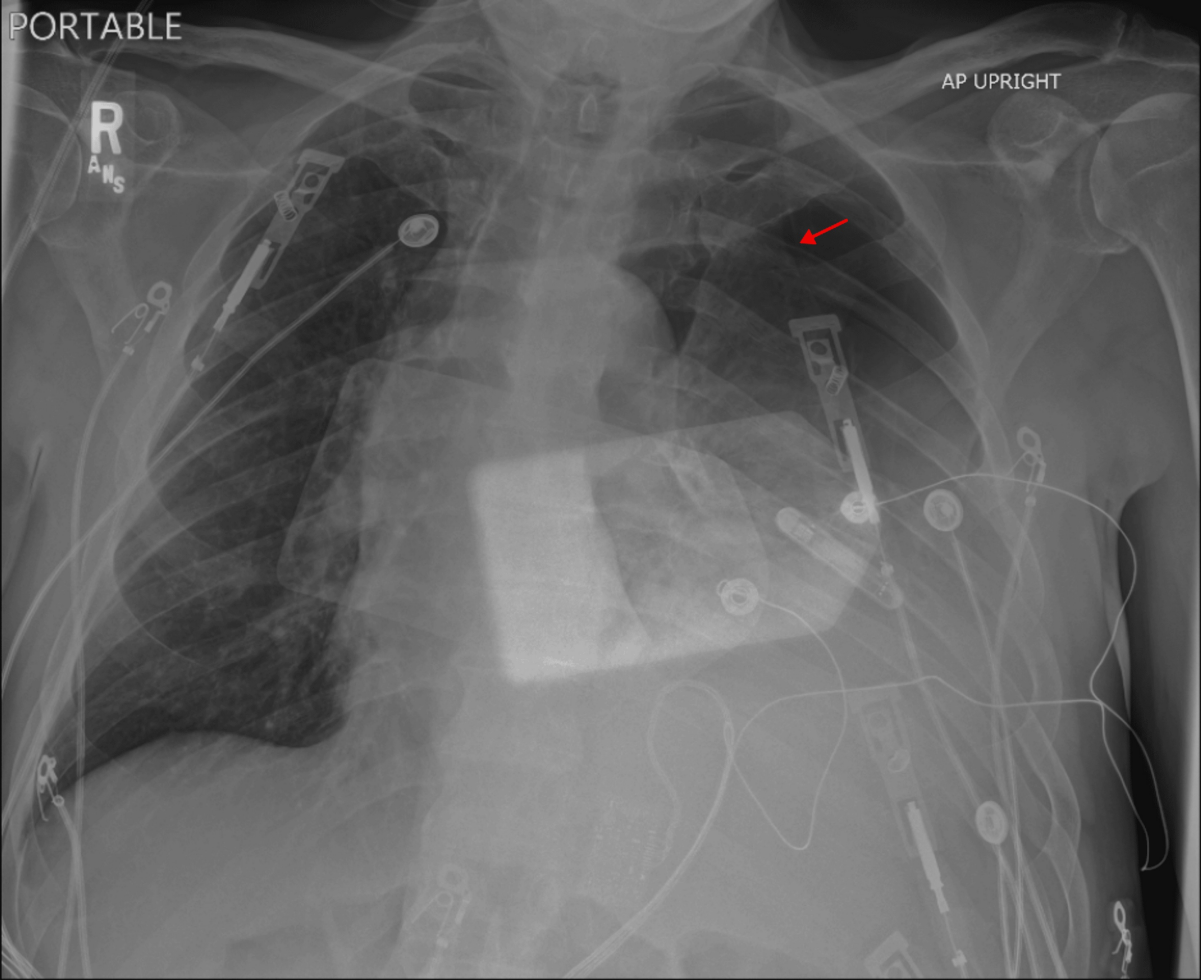
Chest radiography at initial presentation Initial chest radiography showing left-sided hydropneumothorax (arrow) with rightward displacement of the trachea. Overlying defibrillation pads are seen.

**Figure 2:**
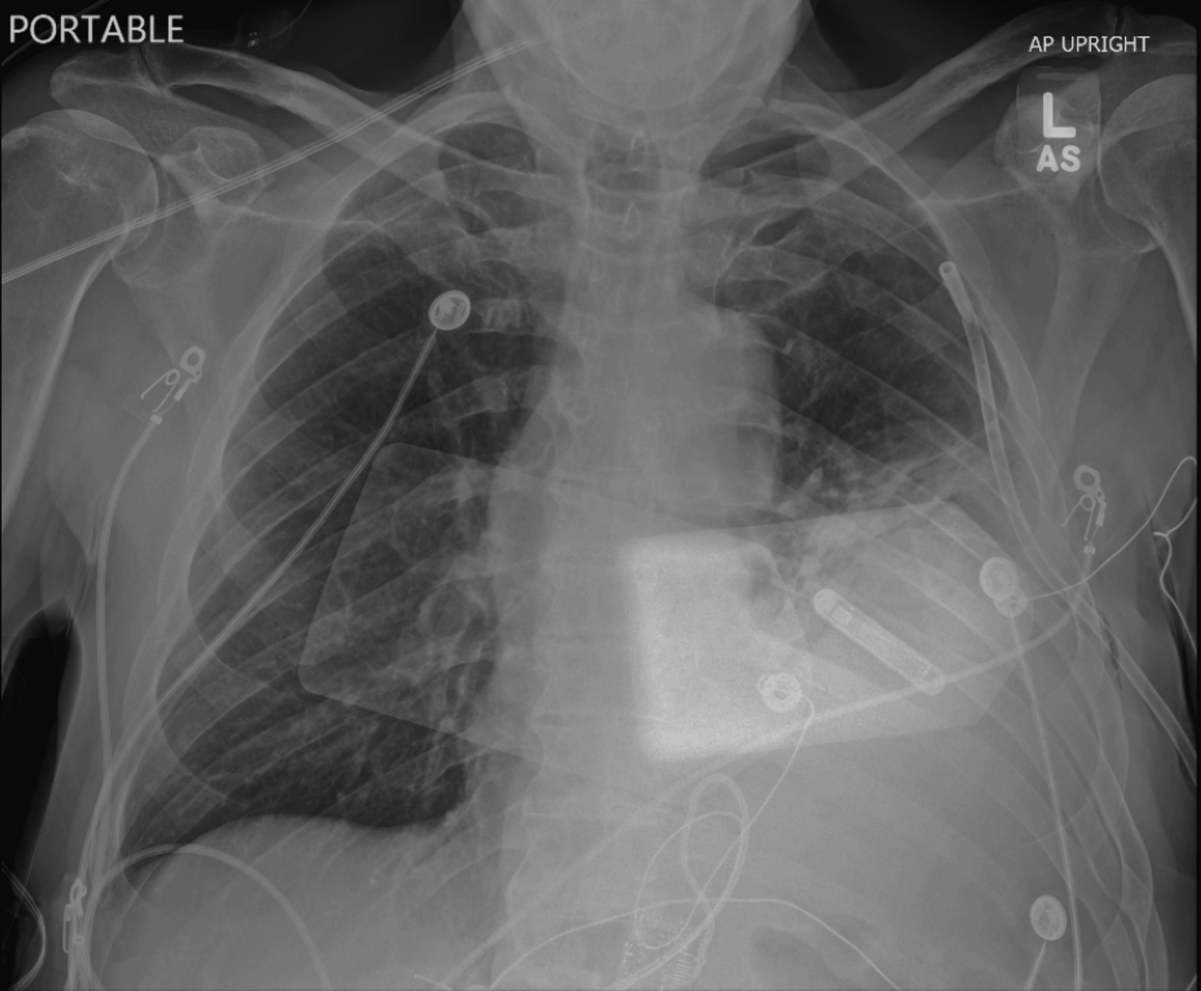
Chest radiography after pigtail chest tube insertion Chest radiography showing improvement in hydropneumothorax and placement of a pigtail chest tube in the left hemithorax with normal tracheal positioning.

**Figure 3:**
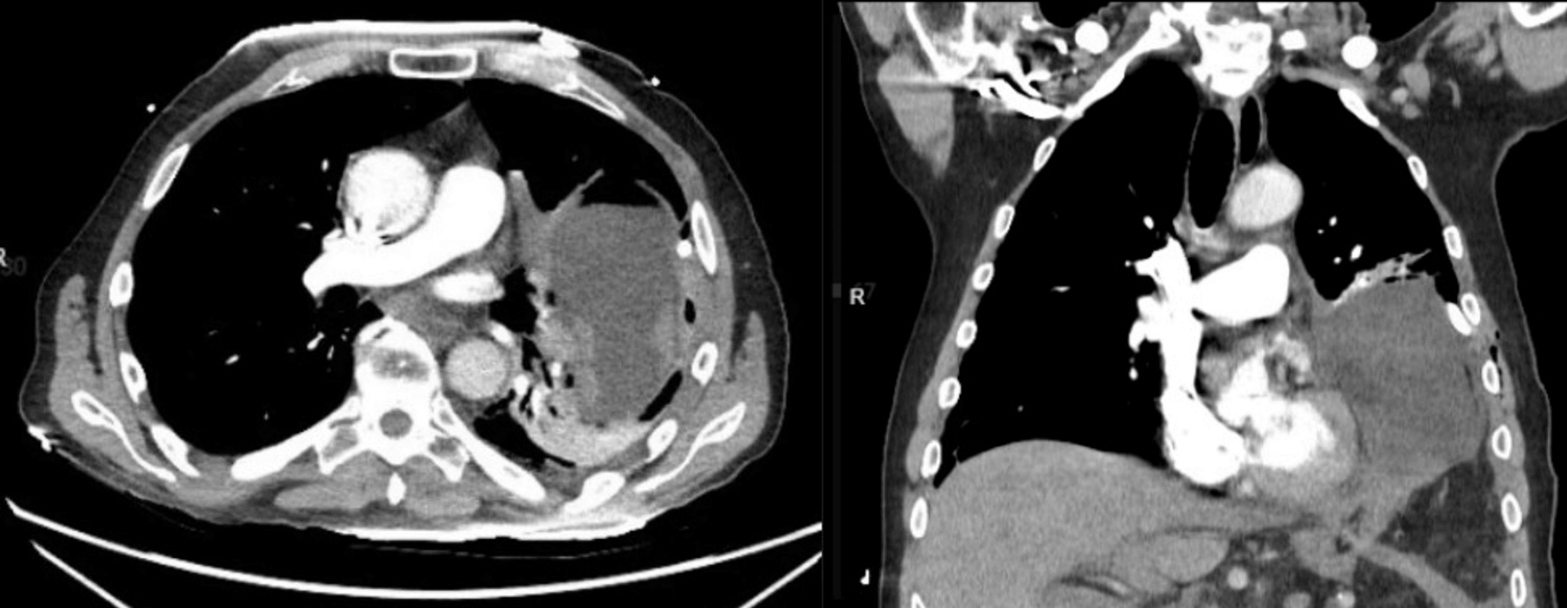
Computed tomography imaging after chest tube insertion showing gastrothorax with adjacent pigtail chest tube CT imaging showing the stomach in the left hemithorax with a chest tube coursing laterally to the stomach.

In the ED, a portable chest X-ray (CXR) (Figure 1) was immediately obtained, which revealed tension hydro/pneumothorax with opacities in the lower left lung field. A chest tube was placed in the fourth intercostal space, which drained over 1 L of bilious fluid and resulted in stabilization of the patient's vital signs. A repeat CXR was obtained, showing improvement in tracheal deviation and hydropneumothorax (Figure 2). Computed tomography (CT) imaging of the chest, abdomen, and pelvis was rapidly obtained (Figure 3). CT revealed development of a large diaphragmatic hernia containing most of the stomach as well as intra-abdominal fat, with a pigtail chest tube coursing laterally and superiorly to the stomach. Fat stranding was noted about the herniated stomach. These images were compared to prior CT imaging from three years prior that revealed a small, fat-containing diaphragmatic hernia. Imaging was also compared to CXR performed 14 hours prior to presentation, which showed the stomach below the level of the diaphragm. 

Additional ED treatment included administration of broad-spectrum antibiotics, fluid resuscitation, and emergent cardiothoracic surgery consultation. The patient was emergently taken to the operating room (OR) for emergent laparotomy, partial gastrectomy, partial omentectomy, and primary diaphragmatic repair. The operative report noted a large area of necrosis about the greater curvature of the stomach with ischemic perforation as well as omental ischemia. The chest tube had not coursed through the stomach. The patient's diaphragmatic hernia was noted to be 10x5 cm and was closed primarily with eight sutures. An esophagogastroduodenoscopy (EGD) was performed, and a nasogastric (NG) tube was placed under direct visualization. Due to gross contamination of the operative bed, a wound vac was placed, and the patient's laparotomy was left open; he was taken back to the OR the next day for washout.

The patient was extubated after washout surgery and weaned off vasoactive medications. His intraoperative cultures grew both Gram-positive and Gram-negative bacteria, and he was continued on appropriate antimicrobials. The patient's pH and lactate normalized postoperatively, and his white blood cell count trended downward. His hospital course was complicated by atrial fibrillation with rapid response, refeeding syndrome, and empyema requiring video-assisted thoracic surgery (VATS). The patient was discharged home on hospital day 14 with a jejunal feeding tube (J-tube). At one-year follow-up, the patient had not had any additional complications from his operation or his hernia and had not required re-hospitalization for any reason. 

## Discussion

This represents a challenging case of a hemodynamically unstable patient presenting with atrial fibrillation, respiratory failure, and obstructive shock, initially thought to be related to tension hydropneumothorax but ultimately secondary to an incarcerated diaphragmatic hernia resulting in ruptured gastrothorax. 

Gastrothorax can present similarly to other conditions that cause obstructive shock and can be difficult to differentiate based on clinical exam. While history helps differentiate between causes of obstructive shock, especially when a hernia may be known, early imaging may be helpful in the recognition of potential anatomical abnormalities that may not be obvious on exam alone [11]. Initial stabilization of an unruptured gastrothorax includes decreasing the pressure exerted by the herniated stomach into the chest with nasogastric tube placement [5]. In any case of hydro- or pneumothorax causing obstructive shock and mediastinal shift, emergent tube thoracostomy is a life-saving procedure. Given the anatomic challenges in this case, chest tube placement into the stomach was a risk. Fortunately, the chest tube was placed in a high enough position that it was able to drain the bilothorax that had resulted from gastric rupture while avoiding placement into or through the stomach. Placement of a chest tube in the fourth intercostal space or higher, as guided by CXR when a diaphragmatic hernia is suspected or known, may help avoid injury to the diaphragm, liver/spleen, or stomach.

Though not specifically outlined in this case, other diagnostic considerations could include the use of bedside ultrasound to identify the diaphragm and note the presence of both air and fluid within the pleural cavity prior to tube thoracostomy. Needle decompression may have initially stabilized the patient due to the component of pneumothorax; however, a large amount of gastric contents would still have remained in the chest and may continue to cause tension physiology.

To the authors' knowledge, there have been no identified cases of ruptured tension gastrothorax presenting to the ED in the medical literature at the time of publication. While gastrothorax is a known mimic of pneumothorax, ruptured gastrothorax further complicates the clinical picture by mimicking a hydropneumothorax. The appearance of pneumothorax with air-fluid levels on CXR in the setting of a known or suspected diaphragmatic hernia may aid in the diagnosis of tension gastrothorax with rupture. Unlike in the case of unruptured gastrothorax, a chest tube should be placed to drain the hydropneumothorax component causing tension, and clinicians should attempt to avoid further injury to the stomach if possible. NG placement was not performed in this case, but could be helpful if the gastric perforation is small. In a stable patient without tension physiology, if ruptured gastrothorax is suspected, CT imaging may be helpful in guiding optimal placement of a chest tube. 

## Conclusions

Diaphragmatic hernia is relatively common, and tension physiology from a ruptured diaphragmatic hernia or spillage of intraabdominal contents into the thorax may be immediately life-threatening. Clinicians should be aware of this entity, especially in cases of known or suspected diaphragmatic hernia. Ruptured gastrothorax may mimic other causes of tension pneumo- or hydrothorax. Identification of gastrothorax as opposed to other causes of hydropneumothorax requires a high level of suspicion and may prevent morbidity if the clinician is able to avoid damage to herniated structures or the diaphragm when placing a chest tube. If the patient's condition allows, CT imaging may help guide safe chest tube placement in the case of ruptured gastrothorax. If a CT is not able to be obtained prior to chest tube placement, placing a chest tube higher than the level of the stomach seen on CXR may help avoid injury to the stomach. Nasogastric tube placement may additionally be helpful in resuscitative efforts by decompressing the stomach in the case of a gastrothorax with a small perforation or small-volume thoracic contents.
